# A national intervention to support frail older adults in primary care: a protocol for an adapted implementation framework

**DOI:** 10.1186/s12877-021-02395-4

**Published:** 2021-08-04

**Authors:** Joanie Sims-Gould, Jacobi Elliott, Catherine E Tong, Anik Giguère, Sara Mallinson, Paul Stolee

**Affiliations:** 1 Centre for Hip Health and Mobility, Faculty of Medicine, University of British Columbia & Vancouver Coastal Health Research Institute, University of British Columbia, Vancouver, BC Canada; 2 School of Public Health Sciences, University of Waterloo, Waterloo, ON Canada; 3 Department of Family Medicine and Emergency Medicine, Laval University, Quebec, QC Canada; 4 Community Health Sciences, University of Calgary / Alberta Health Services, Calgary, AB Canada

**Keywords:** Primary care, Older adults, Frailty, Qualitative research, Study protocol, Implementation science

## Abstract

**Background:**

Older Canadians are high users of health care services, however the health care system is not well-designed to meet the complex needs of many older adults. Older persons often look to their primary care practitioners to assess their needs and coordinate their care. The intervention seeks to improve primary care for older persons living with frailty and will be implemented in six primary care clinics in three Canadian provinces. Presently, more than 1.6 million older Canadians are living with frailty, and this is projected to increase to 2.5 million within a decade (Canadian Frailty Network, Frailty Matters, 2020). The model will include frailty screening, an online portal to expedite referrals and improve coordination with community services, and several tools and techniques to support patient and family engagement and shared decision-making. Our project is guided by the Consolidated Framework for Implementation Research (CFIR) (Damschroder LJ, et al. Implement Scil, 4, 50, 2009). As others have done, we adapted the CFIR for our work. Our adapted framework combines elements of the socio-ecological model, key concepts from the CFIR, and elements from other implementation science frameworks. Nested within a broader mixed-method implementation study, the focus of this paper is to outline our guiding conceptual framework and qualitative methods protocol.

**Methods:**

We will use the adapted CFIR framework to inform the data we collect and our analytic approach. Our work is divided into three phases: (1) baseline assessment of ‘usual care’; (2) tailoring and implementing a new primary care model; and (3) evaluation. In each of these phases we will engage in qualitative data collection, including clinical observations, focus groups, in-depth interviews and extensive field notes. At each site we will collect data with health care providers, key informants (e.g., executive directors), and rostered patients ≥ 70 years. We will engage in team-based analysis across multiple sites, three provinces and two languages through regular telephone conferences, a comprehensive analysis codebook, leadership from our Qualitative Working Group and a collective appreciation that “science is a team sport” (Clinical Orthopaedics and Related Research 471, 701-702, 2013).

**Discussion:**

Outcomes of this research may be used by other research teams who chose to adapt the CFIR framework to reflect the unique contexts of their work, and clinicians seeking to implement our model, or other models of care for frail older patients in primary care.

**Trial Registration:**

U.S. National Library of Medicine, NCT03442426. Registered 22 February 2018– Retrospectively registered.

**Supplementary Information:**

The online version contains supplementary material available at 10.1186/s12877-021-02395-4.

## Background

Like many countries, the Canadian population is aging [1] and older adults living with frailty account for a disproportionate share of public health care spending [2]. Presently, more than 1.6 million older Canadians are living with frailty, and this is projected to increase to 2.5 million within a decade [3]. With a total population of nearly 38 million, there are also 3.75 million family caregivers, or 10 % of the Canadian population, providing support for older adults with age-related care needs [3]. The health care system, however, is not well designed to meet the needs and challenges of an aging population that often experiences numerous, complex health issues [4, 5]. Many older adults require care from multiple providers across multiple settings, but find this care to be overwhelming, uncoordinated and the health system confusing [2, 6–13]. This can lead to inadequate transfers of information [14], medication errors and other adverse events [15, 16], and poorer outcomes [17] for a population that is already vulnerable.

Primary care plays a central role in treatment, coordinating health care, and linking patients to other facets of the health care system [18]; it is often referred to as the “hub” of care or the patients’ “medical home” [19, 20]. In addition to care coordination and treatment, primary care also has the potential to provide preventive care and services for older adults [21]. Older patients tend to look to their primary health care providers (HCPs) to assess their needs and coordinate their care, however physicians report being overwhelmed by patients with increasingly complex and chronic conditions [22].

If we want to improve the overall health care of older adults, in particular those who are frail [23] or pre-frail [24], primary care is the most important site in which to intervene. Recent reviews have found that an effective primary care model for “high-need, high cost” [25] patients requires preventive screening with appropriate follow-up, engagement of patients and caregivers in decision-making supported by evidence, and coordination with other health and social services [25]. These principles will inform our intervention. Aligning with the review conducted by McCarthy and colleagues [25], we aim to implement an evidence-informed intervention to identify, assess, and support older adults living with frailty. This project, ‘*Transforming primary care for older Canadians living with frailty’*, will support patient and family caregiver engagement, stronger care coordination, and the use of technologies to enhance care. Our project takes a mixed-methods approach to data collection, with complementary quantitative and qualitative data collection at each phase of implementation. The qualitative arm of the study, the focus of this manuscript, is informed by ethnographic [26] and process evaluation methods [27, 28]. The central aim of the qualitative component is to document and describe the context that enabled and the processes that enacted change.

The aim of this manuscript to present the protocol for the qualitative arm of the intervention, including an in-depth overview of an adapted and tailored theoretical framework that will guide both qualitative data collection and analysis.

### Theoretical Framework

Our project is guided by the Consolidated Framework for Implementation Research (CFIR) [29]. While there are many implementation frameworks, we chose the CFIR as it is firmly rooted in a thorough review of implementation literature [29] and has been widely used in health research [30–32]. The CFIR has also been used specifically to examine implementation studies in multiple primary care sites [33].

The CFIR consists of five conceptual domains: the intervention, inner setting, outer setting, the individuals involved, and implementation processes [29]. In order to understand implementation, researchers must consider all five of these domains, and how they interact. The intervention refers to the intervention itself, its core elements, and the extent to which it is adapted and tailored to different contexts and sites. The inner setting refers to the context where the implementation occurs (in our case, primary care clinics), and the outer setting refers to the broader economic and socio-political context. The individuals involved include persons delivering, overseeing, evaluating, implementing and/or receiving the intervention. Finally, implementation processes refer to the ‘’active change process” [29] in which the intervention is enacted.

**Fig. 1:**
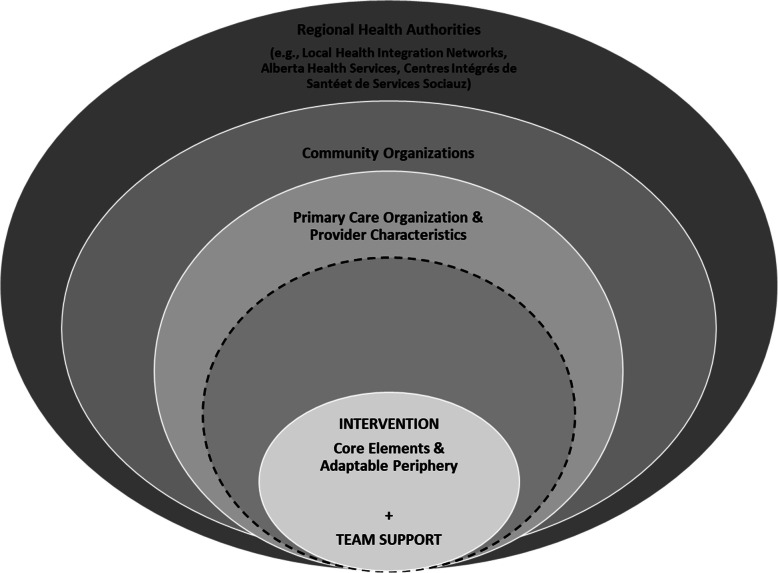
An adapted version of the Consolidated Framework for Implementation Research

As Birken and colleagues [34] note, there are currently more than sixty implementation frameworks, and some projects may find it useful to either combine or adapt models to best address the needs of their particular intervention. As others have done [34–36], we chose to adapt the CFIR. We will use this adapted framework to inform the data we collect and our analytic approach. Our adapted framework combines elements of the socio-ecological model [37] and key concepts from the CFIR [29]. Visually, our adapted CFIR in Fig. 1 reflects the core proposition of the socio-ecological model: in order to understand the function and behaviours of a person, or in our case multiple persons implementing an intervention, we must be mindful of the broader organizational, social and economic contexts. Persons, and interventions, cannot be evaluated and understood separate from the contexts in which they are situated [37].

Our study, further detailed below, is being implemented in three Canadian provinces, therefore we adapted the model to reflect that there are multiple outer settings, *and* multiple layers to each setting. In our adapted model the outer setting consists of two rings, as does the inner setting. The outermost ring represents the different regional health authorities that govern each province: in which the study is taking place - the local health region in Ontario, Alberta Health Services, and the Centres Intégrés de Santé et de Services Sociaux (CISSS)/Centres Intégrés Universitaires de Santé et de Services Sociaux (CIUSSS) in Quebec. In Canada, health care is primarily managed at the provincial level; because the federal government has a very limited role in managing or setting health care policy, our framework focuses on the provinces in the outermost ring. The second outer ring reflects the range of community service organizations that are linked to the primary care sites. In our study this more proximate outer ring reflects the range of community services to which primary care services might refer frail patients, including home and community care, the Alzheimer’s Society, meals on wheels programs, and others. At each clinic site the outer settings will look different, and this must be captured and accounted for. As Damschroder and colleagues [29] recognize, the line between the inner and outer settings is not always clear or firm. One challenge that our intervention seeks to overcome is that patients and caregivers themselves bridge the inner and outer settings; they are the ones who move and often transfer information across these settings and locations of care.

In our model the first inner setting is the primary care organizations in which the model will be implemented. Within this ring, we will consider the values, attributes and characteristics of the primary care clinics. This is consistent with previous uses of the CFIR to assess and understand interventions within primary care [33]. Consistent with the CFIR, we have also added provider characteristics to this inner ring. Scarce attention has been paid to the relationships and interplay between health care organizations and the individual providers embedded in those organizations who are charged with delivering the intervention [29]. We must understand the extent to which individual providers are engaged in the change process, their self-efficacy, and their understanding of and perspectives on the intervention [29].

An additional layer of the inner setting is the patients of the primary care organization, and where applicable, their family caregivers. While the CFIR places ‘patient needs and resources’ and ‘patient characteristics’ within the outer setting, we have chosen to place patients and their characteristics closest to the intervention, as they are the intended recipients of this new model of care. Furthermore, as our intervention explicitly focuses on patient and family engagement in the care planning, it is logical to place patients and families next to the intervention at hand. In the decade since the introduction of the CFIR, there has also been an increased call for meaningful patient engagement throughout the health care sector [38, 39]. The general philosophy of how and where patients should be included in health services work and research has shifted [40, 41], and our inclusion of patients and their caregivers at the center of our framework reflects this evolution.

In their multi-site implementation study, Stetler and colleagues [42] highlight the importance of conceptually including both internal and external facilitators, in their case a study team that was involved in delivering and assessing the intervention. This echoes the PARiHS (Promoting Action on Research Implementation in Health Services) framework and its focus on facilitation of the intervention [43]. This is also reflective of both Wandersman and colleagues [44] and Durlak and DuPres’ [45] frameworks, which both emphasize the central role of support for the intervention. This support can be directly for the intervention (e.g., training), general support (e.g., capacity-building), and support via knowledge translation and synthesis (e.g., provision of evidence-based tools, adaptation of intervention components to meet specific needs) [44]. Drawing on these other implementation frameworks, one of our adaptations to the CFIR was the addition of our study team, represented in the innermost ring and titled ‘team support’. This does not necessarily need to be a research team, but this addition recognizes that within all implementation efforts there are individuals who play a central role in coordination, planning, training, facilitating feedback loops, etc. Here, our team in collaboration with leaders and change agents from the primary care organization will provide training, evidence-based tools, and support to implement the intervention and assess these efforts. The ‘team support’ is intimately linked to the intervention, hence their placement together at the center of the framework. In our previous implementation work [46, 47], we have come to understand that these are two units that must go together: a strong, evidence-based intervention coupled with appropriate, centralized support and facilitation.

#### Methods

### Overview of Study Design & Intervention

Our project is divided into three distinct phases following Sidani and Braden’s [48] recommendations for complex health interventions. The three phases are: (1) Baseline: an assessment of ‘usual care’, (2) tailoring the intervention and implementation: adapting the intervention to each clinic context, training personnel, and introducing the new primary care model, and (3) evaluation: assessing the impact of the intervention on patients and providers. We will collect qualitative data in each of these phases. The primary care model intervention introduces three core elements to each primary care site: use of The interRAI Assessment Urgency Algorithm (AUA), a brief tool that classifies older adults into categories of frailty ‘risk’ [49]; training and the introduction of tailored tools (e.g., decision-boxes) to support patient choice and patient engagement ; and an online, web-based application aimed at streamlining and expediting the referral process and access to community resources and services (e.g. meals on wheels, home and community supports). More details on the intervention can be found in Stolee et al. [50].

### Setting & Context

We are implementing this intervention in six primary care clinics, both urban and rural, in three Canadian provinces: three clinics in Southwestern Ontario, one clinic in Quebec and two in Alberta. Primary care HCPs (including physicians, nursing, and allied health professionals) at each clinic will participate in all phases of the study. In Canada, universal health care is guaranteed to all Canadians via the federal Canada Health Act [51]. While care is guaranteed at the national level, each of the ten Canadian provinces and three territories are responsible for delivering care. Each province organizes health care somewhat differently, producing a checkerboard of different systems, policies and practices [52]. The three provinces in which we are conducting this study each have different funding mechanisms, policies, organizational structures, and information technology systems (e.g., different electronic medical records systems) that impact primary care.

### Sample Selection & Recruitment

To understand the impact of the intervention on patients, family caregivers, and providers, per the inner rings of our conceptual framework, we will conduct observations, interviews and focus groups with a wide range of stakeholders. All participants will be provided with consent forms, approved by our respective university research ethics boards, prior to data collection. Participants who take part in data collection at multiple time points (e.g., a health care provider who completes a focus group at baseline and after the intervention) will be re-consented at each instance. We aim to recruit 6–8 patients and health care providers from each study site. This sample size is appropriate for qualitative research. If saturation is not reached, additional participants will be recruited. Recruitment of participants is currently in progress.

#### Older adult participants

Eligible older adult participants are individuals aged 70 years and older, and who have been a rostered patient at one of our clinical sites for more than six months. Patients living with dementia and their caregivers will also be eligible to participate. Patients will be excluded if they do not speak English or French. At both baseline and during the intervention, the study will recruit 70 older adult patients from each of the six sites for quantitative assessments (see [50] for more detail). At each site we will aim to interview eleven of these participants, for a total of approximately 66 patients at baseline and 66 patients at intervention. On the consent form for the quantitative assessments, participants will be asked to indicate if they would be willing to participate in a follow-up interview. Research assistants will telephone those who indicate their willingness to complete an interview. We will use participants’ survey data (e.g., their self-rated health, number of chronic conditions and frequency of emergency room visits) to purposively sample [53] interview participants who represent the spectrum of patients in the broader study.

#### Health care provider participants

Eligible health care providers (HCPs) are any staff members involved in the delivery of health care to older patients at any of our study sites. Participants may include nurses, nurse practitioners, dieticians, administrative staff, physicians, and other clinical staff. Health care providers will be excluded if they do not speak English of French. HCPs will be recruited by the executive directors or managers at each site, and data collection with these participants will take place during paid work hours. We anticipate recruiting approximately five to seven HCPs per focus group.

#### Key informant participants

We anticipate working with key informants throughout the study, to plan, tailor and implement the intervention. Key informants are individuals invested in the study from an administrative perspective, and may include office administrators and managers, executive directors, etc. Key Informants will be recruited via a direct email from our research team. These are persons who have been involved with the study from the outset.

### Data Collection Procedures

**Fig. 2:**
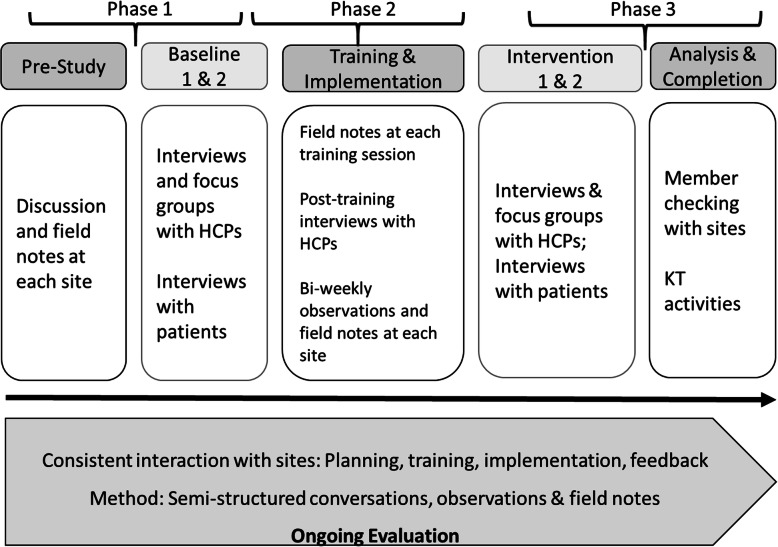
Overview of data collection

Our data collection is also aligned with Sidani and Braden’s [48] three phases. We have mapped our data collection onto these phases in Fig. 2. All interviews will be semi-structured, digitally recorded and conducted in private locations of the participants’ choosing (e.g., a clinic office). Master’s and PhD trained qualitative health researchers will conduct the interviews, using semi-structured interview guides (detailed below, see Supplementary Files 1 & 2). Where appropriate, we may also utilize telephone interviews. We anticipate that interviews with HCPs and patients will last between 30 and 60 min. All focus group interviews will occur in clinic offices, will be digitally recorded, and will be conducted by a Master’s or PhD trained research team member with prior experience leading focus groups and who will have familiarity with the local context, clinic and intervention components. Focus groups will only be conducted with HCPs, and each focus group session will begin with a reminder about confidentiality and privacy within the context of a shared focus group discussion [54]. We anticipate that focus groups will last 45 to 60 min, and these will take place during paid clinical time. Participants will not receive honoraria.

As outlined at the bottom of Fig. 2, and in all phases, we anticipate that a significant portion of our data collection will consist of observations, field notes, and interviews. Research team members, including experienced co-investigators, their trained graduate students, and research associates will conduct observations at a range of implementation events and activities (training sessions, key informative interviews, HCP focus groups, clinic observations during implementation, research team meetings, etc.). Team members will compose field notes both *in situ*, during the observation, and within 24 h of completing the observation. Our approach to conducting observations and writing field notes is informed by the ethnographic tradition, and specifically the work of Emerson, Fretz, & Shaw [55]. Field notes may be taken down on paper or digitally, with the expectation that field notes will be digitally transcribed before the end of each phase, to allow for ongoing analysis and shared learnings. Our team has previous experience conducting a national, multi-site ethnographic study [12] and we have adapted a tested observation guide from that experience (see Supplementary Materials). Where appropriate, some field notes (e.g., from a research team meeting) may take a narrative, unstructured format.

We will collect data for each of these phases using a combination of clinical observations, meeting and implementation observations, focus groups with HCPs, and interviews with both patients and other key informants. Preliminary interview and focus group guides have been developed by our team’s Qualitative Working Group and have been reviewed by all co-investigators (as detailed in Supplementary Files 1 & 2). In each guide we have used the CFIR and our adapted version of the CFIR to guide the questions we will ask and concepts that we will probe. Sample questions are outlined in Table 1, and preliminary interview guides are available in the Supplementary Materials. We have labelled these as preliminary guides, recognizing that qualitative research is an iterative process; initial analyses, observations and feedback will may alter the sorts of questions that we ask [56].

**Table. 1:** Aligning Data Collection with the adapted CFIR Framework

Framework Component	Type of Data Collection	Sample Question
**Regional Health Authority**	Focus groups with HCPs & Key Informant Interviews	How are people connected to your services? What do you see as some of the barriers/facilitators to care coordination for your older patients?
**Community Organization**	Interviews with HCPs & Patients	Can you tell me about your current process for referrals to community services? Can you walk me through the process of accessing the community services you were referred to?
**Primary Care Organization & Provider Characteristics**	Focus groups with HCPs and Key Informant Interviews	Can you walk me through your process when a frail, or potentially frail, older adult comes in this office? Tell me about your role? In your opinion, what are the best learning strategies for improving your clinical skills with regard to the use of these tools with older patients?
**Patient & Caregiver Characteristics**	Interviews with patients	What questions did you have about your health? How involved did you feel in discussions/decisions made related to your care? How would you rate your current health?
**Central Support**	Interviews and/or questions following training sessions with HCPs Focus Groups with HCPs In-depth interviews with research team members who supported the implementation.	Rate your knowledge on the AUA, Caredove (web-based referral platform), etc. Are there any aspects of the intervention that you require further training on? Further clarification? In the past, how have new programs/tools/resources been introduced (implemented) at this clinic? What worked? What didn’t? How do you think the intervention is working in your clinic? What’s working? What isn’t? Which aspects of implementation required the most of your time/support/expertise? If another research team wished to enact a similar intervention and evaluation in primary care, what advice would you give them?

### Multilingual Data Collection & Analysis

Quebec is a predominantly French-speaking province, while Alberta and Ontario are predominantly English-speaking. In Quebec, training, implementation, and data collection will be conducted in French. In order to work collectively, our team-based work to-date has been conducted in English. Each site will conduct their site-specific analyses in the language of data collection. We anticipate that future team-based analyses of our qualitative data will be done with a combination of French and English coding. Final research reports and manuscripts will include data excerpts translated into English by multilingual research assistants and/or the bilingual co-investigators. Consistent with the process used in Tong et al. [57], we will have a second bilingual research assistant check 10 % of the translated texts for consistency. If significant issues are flagged in these translation reviews, we will engage in a more formal forward-and-back translation process, as advocated by the World Health Organization [58] for multilingual data collection and analysis.

### Team-Based Qualitative Analysis across Multiple Sites

Team-based qualitative analysis produces both challenges and opportunities. By including the voices and disciplinary perspectives of multiple researchers, team-based analysis can enrich the interpretation of the data and enhance overall rigour; however, it also requires thoughtful planning, ongoing communication and leadership [59]. In our study we will have qualitative teams engaging in data collection and analysis in each province. As Guest & MacQueen [59] note, team-based, multi-site qualitative analysis requires leadership; at the outset of this study we formed the Qualitative Working Group to establish protocols and coordinate data collection and analysis across all sites. Throughout the study, the qualitative working group will meet annually in-person/via webinar and will meet quarterly via teleconference. We also anticipate regular telephone meetings and the sharing of documents via email. Data at all sites will be transcribed by either trained research assistants or professional transcription agencies. Data will be blinded, with all potentially identifying information replaced with codes. These codes may include [patient name], [clinic name], [physician’s name], etc. All participant names will be replaced with unique numeric identifiers, and blinded transcripts will then be uploaded into NVivo 12 (QSR International, 2019) for analysis. To coordinate data analysis across the provincial sites, the working group will develop a codebook that will guide thematic coding of the data, by clearly identifying code names and definitions [60]. Codebooks help facilitate consistent coding across sites, particularly when the sheer volume of data requires multiple coders. Based on our preliminary work with the implementation sites and through several team discussions within the working group, we have developed an *a priori* coding structure. This *a priori* coding structure, in Table 2, includes concepts from the CFIR and our adapted CFIR. We anticipate that there will be different codes and different salient themes for each region and clinic site, as every context is different. This initial framework is not prescriptive, but rather provides a common starting point, with the expectation that implementation, coding and analysis will occur slightly differently in each site.

**Table. 2:** * A priori* coding structure, based on concepts in the original and adapted CFIR

Coding Node	Sub-nodes
**Patient & Caregiver Characteristics**	• Patients • Caregivers
**Adapted CFIR**	• Community Organizations o Community Services o Referral process
	• Primary Care Organization o Attributes, Values, Characteristics o Clinical Description ■ Appointment Time ■ Assessment Tools o Communication Practices ■ Patient Engagement Practices
	• Regional Health Authority o Funding Mechanisms o Policies
	• Central Support and Synthesis Translation o Guidance for Implementation ■ Challenges with Implementation ■ Successful Past Implementation ■ Suggestions for Delivery of Implementation o Intervention Characteristics o Intervention Training ■ Mode of Training ■ Organization of Training o Support by Project Team
**Other**	

### Multi-Site Considerations

As a national team, with data collection occurring in three provinces and two languages, we have been especially mindful of the procedures and policies that need to be in place to do this work. With lead researchers at three universities, we acquired ethics approval from each institution. We also worked with the university liaison offices at each institution to develop data sharing agreements, which will allow us to move data as needed between sites for analysis. In order to move and share data between sites, we will use a secure, university-approve online portal (www.sharepoint.com) for our team. Leads at each of the provincial sites have access to this portal, in addition to key team members (e.g., research coordinators and graduate students who will be involved in data collection and analysis.

### Site-Specific Tailoring and Adaptations

In the CFIR, Damschroder and colleagues [29] posit that multi-site interventions will have core elements, but that they will also have flexibility across sites to make decisions that are reflective of distinct contents and different partners. The CFIR states that interventions will have ‘core components’, that must be consistent across all sites, and an ‘adaptable periphery’, which allows for appropriate modifications as necessary [29]. We have adopted this philosophy for both our implementation and for the study itself. We anticipate that each clinical site will adapt and modify the intervention in a manner that makes the most sense for their practice. Similarly, we anticipate that each provincial research team will adapt and modify data collection tools and intervention training; for example, we will have a core set of questions for interviews with patients but expect that some sites may add questions that are specific to their context.

### Seniors Helping as Research Partners (SHARP)

Our research team has involved older adults and their caregivers in all aspects of our research and knowledge translation work. In 2013, we launched *Seniors Helping as Research Partners* (SHARP; https://uwaterloo.ca/ghs/sharp; also described in Table 3), a group of more than 60 older adults, with whom we have built a collaborative partnership, following evidence-based principles for engaging older adults and their caregivers in health care research [61, 62]. To date, the SHARP group has reviewed the study proposal and the interview guides that we intend to use with older patients, and has provided feedback on all of the tools (e.g., decision boxes, web-based applications) that form part of the intervention. We anticipate that the SHARP members will also be involved in additional research and knowledge translation activities as the study unfolds.

**Table. 3:** The SHARP Group

*Seniors Helping As Research Partners*
• 60 + members
• Live in various medium and small cities in Southern Ontario, Canada
• Participants live in a range of settings, including community-dwelling, retirement communities and assisted living facilities
• Members include men and women aged 65 to 94, majority are Caucasian
• Some have served with the SHARP since 2013. Participation ranges from eight to one year, and recruitment for SHARP is ongoing
• Some speak from the perspective of an older patient, while others focus on their perspectives as family caregiver

### Strategies for Rigour

To promote trustworthiness of the findings, we will adhere to Lincoln and Guba’s [63] criteria: credibility, dependability, confirmability and transferability. Credibility will include member checking with key stakeholders and peer debriefing amongst the research team, including regular multi-site teleconferences to ensure credibility and consistency across sites. We will establish dependability through the use of triangulation (varied data collection methods such as interviews, observations, and field notes). An audit trail [64] and various iterations of the evolving codebook [60] will provide confirmability that enables another reader or researcher to follow the progression of events in the study and analytic decisions. We will have a global audit trail that overviews the entire study, while each site will also administer an audit trail for their provincial sites and analyses. Within the global audit trail, we will create a specific section that tracks any implementation and/or analytic modifications that are site-specific and are tailored to either one site or one province. We will achieve transferability through “thick description” of the findings that enables those interested in making a transfer of the findings to other contexts or another time. This thick description will largely draw on implementation field notes. We will use the TIDier (Template for Intervention Description and Replication) checklist when reporting on our intervention [65]; in the reporting of our qualitative analysis and results we will use a combination of the COREQ (Consolidated Criteria for Reporting Qualitative Research) [66] and SRQR (Standards for Reporting Qualitative Research) [67] guidelines. Having these checklists in place at the outset of data collection will ensure that we capture and record sufficient detail for our reporting.

### Knowledge Translation and Dissemination Plan

Most Canadian agencies funding research, including the funders for this project, expect a high-degree of knowledge translation to take place. We will engage in ongoing, integrated knowledge translation and exchange [68], with the aim of sharing both the outcomes of the research (i.e., the ‘results’) and lessons learned and best practices for doing this type of team-based research in a primary care setting. Integrated knowledge translation posits that decisions-makers and researcher work collaboratively throughout the research project [68], with the expectation that these established partnerships will facilitate moving research findings into practice. We have been working with decision-makers within primary care since the inception of this project. We anticipate that our knowledge translation outputs will include: academic and community-based presentations; peer-reviewed, open access publications in relevant and highly visible journals; evidence and policy briefs on partner, funder and university websites; evidence briefs for provincial Ministries of Health; and plain-language tools or guides related to our implementation and evaluation processes. We will also host a large knowledge translation event near the end of the project, to share our results with partners, health care providers, research participants, members of SHARP, local and provincial policy-makers, and student trainees.

 This study has received ethics clearance from a number of institutional research boards, including those at the Universities of Calgary, Laval and Waterloo.

## Discussion

Consistent with current implementation science practice [34] we have combined and modified elements of the CFIR and other appropriate models to frame our study. We have mapped our data collection and analytic tools onto the CFIR; this framework and approach will ensure that we capture comprehensive data that truly speaks to the implementation process, and reveals what does, and potentially what does not, render positive changes for frail older adults in primary care. In concluding the original CFIR article, Damschroder and colleagues [29] argue that ultimately the utility of their model will be determined by a series of questions, including “Does the CFIR promote comparison of results across contexts…?”. In implementing across six primary care sites, in three provinces and two languages, our results will help to answer this question.

### Strengths and Limitations

As with any implementation project there are always unforeseen challenges at multiple levels. Through our qualitative approaches we will note contextual factors (such as a change of government) that might influence implementation.

Informed by ethnographic techniques [26], process evaluation methods [27, 28] the CFIR [29] and integrated knowledge translation [68], we are cognizant that partnering with decision-makers and key stakeholders is vital to the success of this implementation. Gagliardi et al. [68] have noted that researchers must be more mindful of systemically planning and documenting their design and implementation, particularly work with stakeholders. We have developed this protocol with this recommendation in mind, and this level of engagement will be a strength of this work. Also, working across multiple provinces and languages will provide essential practical insights into conducting national- and international-level interventions the require working with multiple site and teams, providing meaningful central support at a distance, ensuring rigour and fidelity across linguistic barriers, and adapting interventions to a range of diverse clinics operating with distinct telecommunications systems, different provincial-funding schemes, and constant health system restructuring [69]. Working across multiple languages, diverse sites, and with a range of scientists and practitioners, a strength of this research is the approach to science as a "team sport" [70]. 

## Conclusions

While the quantitative analysis of the intervention [50] will tell us about the relative successes of the intervention, the qualitative data outlined here will allow us to understand the implementation processes [27, 29] that enacted change, and provide a robust description of the varied contexts in which changes were made. Irrespective of the outcomes, engaging in and systematically documenting this research will provide important insights into how to implement new models in primary care, particularly complex interventions with multiple components being adapted for multiple sites, across several regions, and in multiple languages. This will make a significant contribution to the implementation science literature, and continued efforts to improve primary health care for an aging global population.

## Supplementary Information

**Additional file 1**: HCP Interview Guide. The semi-structured interview guide/focus group guide used for data collection with health care providers.

**Additional file 2:** Patient Interview Guide. The semi-structured interview guide used for data collection with patients.

## Data Availability

Not applicable for study protocol.

## Abbreviations

**CFIR**: Consolidated Framework for Implementation Research.
**HCPs**: Health care providers.
**CISSS/CIUSSS**: Centres Intégrés de Santé et de Services Sociaux (CISSS)/Centres Intégrés Universitaires de Santé et de Services Sociaux.
**PARiHS**: Promoting Action on Research Implementation in Health Services.
**AUA**: interRAI Assessment Urgency Algorithm.
**SHARP**: Seniors Helping as Research Partners.
**TIDier**: Template for Intervention Description and Replication.
**COREQ**: Consolidated Criteria for Reporting Qualitative Research.
**SRQR**: Standards for Reporting Qualitative Research.
