# Supplementary material for: A national intervention to support frail older adults in primary care: a protocol for an adapted implementation framework
Source: BMC Geriatr. 2021 Aug 4;21:453. doi: 10.1186/s12877-021-02395-4 (PMC8336337; doi:10.1186/s12877-021-02395-4)
Supplement: Supplementary file 1 — Additional file 1: HCP Interview Guide. The semi-structured interview guide/focus group guide used for data collection with health care providers. [file 12877_2021_2395_MOESM1_ESM.docx]

# **Individual/Focus Group Interview Questions with Health Care Providers**

***Introduction Script:*** *Thank you for agreeing to participate today. As described in the Letter of Information, with your permission we will be audio recording this session. Your names will not be associated with the data.*

*We have a series of questions related to understanding the current process here at [clinic name] as well as questions about the implementation of a care coordination intervention for older adults living with frailty. Do you have any questions before we get started?*

***PART 1: BACKGROUND AND CURRENT PRACTICE***

*We want to get some background information about you, the clinic, and your practises and then explore a few more things around patient and family engagement in care planning and decisions. OK?*

1. **Tell me about your role?**
   1. *How long have you been in this role? What’s do you do? Who is in your team? Who are your clients (proportion older)? Do you have any specialist role in relation to older people (e.g. oncall in LTC)?*
2. **Focusing on your older clients:**
3. How long does an appointment usually take?
   - - **PROBE: How long is an appointment that you would describe as ‘long’?**
     - **PROBE: How long is an appointment that you would describe as taking a ‘normal time’, or taking a ‘short time’ with older patients?**
   1. In what situations would you spend more time with a patient?
      - [PROBE around identifying and referring for community services?]
4. **We’d like to know more about the kind of assessment tools you may use with your older patients to assess their health risks and/or care needs.**
   1. Can you tell me about any standardized tools you use routinely or occasionally **with patients who come to visit you in this setting? [the MDs who we will recruit work in other setting (homecare, hospital, and sometimes long-term care settings or residential homes)]**
      - PROBE for details: Which ones, when, and what for? Why that tool? What do you do with the information?
   2. Are you familiar with any other standardized assessments **in this population?**
      - **PROBE: Why not using?**
5. **We are interested in patient and family engagement in care decisions and want to know more about how health care providers manage that in daily work with older people and families.**
   1. Are you familiar with shared-decision making ideas? (probe around personal experience, learning and practice and any details on practice or network context, team, etc )
   2. In general, how do you share information about treatment or other support options with patients and their caregivers and how you get their feedback on goals and preferences to allow them to make a decision?

*Probe: how do you make decisions about referrals/treatment plans with patients? Are there any specific approaches you use?*

*Are there limitations/challenges to engagement? Discuss what works.*

- 1. Tell me about any shared decision-making tools or resources that you are using, or have tried in the past, to help engage your patients in care planning? (Probe: what, when, why, what happened?)

1. **We are also interested in how you connect with community services. Are you currently referring older patients to community services?**
   1. How do you currently make that referral? (e.g. fax; telephone; self-refer)
   2. Do you currently collaborate/ communicate with specific community services/ agencies that provide services to your older patients?

**Field Notes:**

**Date of Interview/Focus Group:**

**Length of Interview/Focus Group:**

**If focus group, number of participants and setting:**

**Interview/Focus Group completed by:**

**Field notes completed by:**

**Notes: (high-level themes, key words, key quotes, any areas of inquiry that seemed especially interesting, uncomfortable, questions that worked/didn’t work, questions or probes that we need to add/consider, etc.)**
