# Supplementary material for: A national intervention to support frail older adults in primary care: a protocol for an adapted implementation framework
Source: BMC Geriatr. 2021 Aug 4;21:453. doi: 10.1186/s12877-021-02395-4 (PMC8336337; doi:10.1186/s12877-021-02395-4)
Supplement: Supplementary file 2 — Additional file 2: Patient Interview Guide. The semi-structured interview guide used for data collection with patients. [file 12877_2021_2395_MOESM2_ESM.docx]

# **Individual/Focus Group Interview Questions with Patients**

***Introduction Script****: Thank you for agreeing to participate today. With your permission, we will be audio recording this session. Your name will not be associated with the data.*

*I am going to ask you some questions about your current experiences at your health care clinic. There are no right or wrong answers: we are interested to hear what you think.*

*Before we start I just want to remind you that taking part is completely voluntary. Let me know if you want to skip a question or if you want to stop the interview. Do you have any questions for me?*

**1. Opening question:** Would you like to tell me a bit about yourself? (probe for age, where they were born, current living situation, family, etc. Just keep this open and let it set the stage for a more meaningful conversation).

**2. Current care practices, including referrals and integration:**

- We would like to understand your experiences at your health care clinic…
  - Core Questions:
    - - What conditions are you currently managing?
      - Can you walk me through a typical appointment?
      - Does anyone attend appointments with you? (If yes, what is their role?)
      - Who do you receive care from within your clinic, a family physician? Any others?
      - Do you go anywhere else for health care? (probe for physio, regular bloodwork, specialists, etc.)
      - Are there services that you need but are currently not receiving? Why do you think that is?
      - Have you been referred elsewhere in the last year? Can you tell me about that process? (probe for: who sent the referral, how was the appointment information sent back to you? A call? From whom?)
      - [If referred]- what is the communication like between your family doctor and the specialisists. Do you feel like everyone is one the same page?
      - How often have you had to repeat ‘your story’, your description of your health? What do you think we could do to change this?
- Probes:
  - - - Probe for any discussions around receiving home care, and coordinating with home care services
      - Are there any other services or programs at the clinic that you take part in? (e.g, blood pressure clinics, fitness classes, courses, etc.)

**3. What does “good primary care” look like:**

- What does a ‘good clinic’ look like?
- Is there anything that could be done to improve your current clinic?
  - (**If no**, what makes your clinic an example of excellent primary care for older adults?)
- How long is your typical appointment? Are you limited to one ‘issue’ per appointment?
- For people who seem to be long-term patients: how has the clinic changed in the last 5-10 years?

**4. Patient engagement & decision-making:**

- Do you feel like you have “a say” in your health care decisions/planning? What makes you say this? Can you give me an example of when you had “a say”/”input”?
  - If person said ‘yes’ above to someone going to appointments with them, probe for decision-making with caregivers. When making a decision with your health care provider, how involved is [name] in that conversation?
- When making an important health care decision, how do you do this?
  - Probes: do you go home and think things over? Who do you consult? Do you do your own research? If yes, how do you do this research? Do you receive education materials/information to help you make decisions? Do you make a follow-up appointment? How does the final decision get made?
- Have you ever had an instance in which you did not agree with your doctor’s (or NP or other clinician?) recommendation or assessment? [If yes, can you tell me about that?]
- Sometimes we hear that older adults are less likely to question physicians compared to other generations. Do you think this is true? Why is this?
- Who do you like the best at your current clinic? What makes them a good health care provider?
- Does your provider know what matters most to you?
- What do you do to look after yourself?

**5. Other topics and wrap-up questions:**

- Do you think that men and women approach health care and family doctors differently? What makes you say that?
- Is there anything else that you would like to tell me about your clinic?
- Is there anything else that you would like to tell me about health care for older adults?

**Field Notes:**

**Date of Interview:**

**Length of Interview:**

**Interview completed by:**

**Field notes completed by:**

**Notes: (high-level themes, key words, key quotes, any areas of inquiry that seemed especially interesting, uncomfortable, questions that worked/didn’t work, questions or probes that we need to add/consider, etc.)**
